# Computational modeling method to estimate secondhand exposure potential from exhalations during e-vapor product use under various real-world scenarios

**DOI:** 10.1007/s11739-022-03061-2

**Published:** 2022-09-01

**Authors:** Jeffery S. Edmiston, Ali A. Rostami, Qiwei Liang, Sandra Miller, Mohamadi A. Sarkar

**Affiliations:** Center for Research and Technology, Altria Client Services LLC, 601 East Jackson Street, Richmond, VA 23219 USA

**Keywords:** E-vapor, ENDS, E-cigarettes, Secondhand exposure, Nonusers, Bystanders, Computational model predictions, Real-world scenarios, Nicotine, Formaldehyde

## Abstract

Potential secondhand exposure of exhaled constituents from e-vapor product (EVP) use is a public health concern. We present a computational modeling method to predict air levels of exhaled constituents from EVP use. We measured select constituent levels in exhaled breath from adult e-vapor product users, then used a validated computational model to predict constituent levels under three scenarios (car, office, and restaurant) to estimate likely secondhand exposure to non-users. The model was based on physical/thermodynamic interactions between air, vapor, and particulate phase of the aerosol. Input variables included space setting, ventilation rate, total aerosol amount exhaled, and aerosol composition. Exhaled breath samples were analyzed after the use of four different e-liquids in a cartridge-based EVP. Nicotine, propylene glycol, glycerin, menthol, formaldehyde, acetaldehyde, and acrolein levels were measured and reported based on a linear mixed model for analysis of covariance. The ranges of nicotine, propylene glycol, glycerin, and formaldehyde in exhaled breath were 89.44–195.70 µg, 1199.7–3354.5 µg, 5366.8–6484.7 µg, and 0.25–0.34 µg, respectively. Acetaldehyde and acrolein were below detectable limits; thus, no estimated exposure to non-EVP users is reported. The model predicted that nicotine and formaldehyde exposure to non-users was substantially lower during EVPs use compared to cigarettes. The model also predicted that exposure to propylene glycol, glycerin, nicotine and formaldehyde among non-users was below permissible exposure limits.

**Supplementary Information:**

The online version contains supplementary material available at 10.1007/s11739-022-03061-2.

## Trial registration

ClinicalTrials.gov Identifier: NCT04881942, first posted 11/05/2021.

## Introduction

Electronic vapor products (EVPs) are a growing segment of smoke-free products in the category of products containing tobacco-derived nicotine. These devices may offer adult tobacco consumers a reduced-risk product compared to conventional, lit-end cigarettes and play an important part in tobacco harm reduction [1–3]. EVPs deliver nicotine in an aerosol that has a very different composition from conventional cigarette smoke [4–6]. Many of the chemicals generated from the combustion of tobacco are harmful [7, 8]. In contrast, heating an e-liquid consisting of carrier constituents (e.g., propylene glycol or glycerin), nicotine, water, and flavors generates far fewer harmful chemicals in much lower levels than the burning of tobacco [4, 9]. However, EVP aerosols may contain some chemicals like carbonyls, volatile organic constituents, and metals [5, 10]. The inhaled EVP constituents are delivered to the user’s mouth, throat, and lungs during use and may be released into the environment during exhalation. Understanding the potential exposure of EVP exhaled constituents to non-users of EVPs is an important part of assessing the EVP’s overall potential for harm reduction. However, few studies have assessed the levels of constituents contained in the exhaled breath of EVP users and air levels under various real-world scenarios to estimate potential exposure to non-users [5, 11, 12].

Results from quantitative and qualitative studies show a wide range of constituents in the aerosols of EVPs [13, 14]. Most studies use smoking machines to generate and directly collect the EVP aerosol [15]. Data from such studies do not consider the uptake of the aerosol by the user. While sidestream smoke from a smoldering cigarette and smoker exhalations are both non-user exposure sources from a conventional cigarette, the aerosol exhaled into the environment by the user is the only potential source for secondhand exposure during EVP use [16]. Accurate estimates of the level of constituents within aerosols exhaled after EVP use can be used to model potential exposure to non-users under a variety of space and use conditions.

The purpose of this study was to characterize the level of selected aerosol constituents in exhaled breath after use of four different e-liquids in a cartridge-based EVP. In addition, we used the exhaled breath concentrations to model the constituent levels in the air of several indoor space configurations and usage scenarios to understand the potential for secondhand exposure to non-users. As the EVP segment continues to grow, the models used in this research can provide insight into how non-users may be exposed to EVP constituents in different space and real-world use scenarios.

## Methods

### E-vapor products

The EVPs were unbranded versions of MarkTen^®^ XL (Nu Mark LLC, Richmond, VA). The test products are no longer sold commercially and have been discontinued since December 2018. Each test product consisted of a standard battery and cartridge, whereas sham EVPs had inactivated batteries and empty cartridges. Subjects used the assigned e-liquid on each study day:

- Test Product 1: MarkTen.^®^ XL Fusion (2.5% nicotine by weight; NBW)
- Test Product 2: MarkTen.^®^ XL Bold Classic (4.0% NBW)
- Test Product 3: MarkTen.^®^ XL Winter Mint (3.5% NBW)
- Test Product 4: MarkTen.^®^ XL Bold Menthol (4.0% NBW)

The solution weight in an unused cartridge was ~ 900 mg for all four e-liquids.

The seven analytes were measured for each e-liquid using a puffing machine with 20 puffs/cartridge using a 5-s puff duration and 55-cm^3^ puff volume at 30-s inter-puff intervals (Supplementary Table S1).

### Exhaled breath collection study design

This study was an open-label, four-way crossover study designed to measure nicotine, propylene glycol, glycerin, menthol, formaldehyde, acetaldehyde, and acrolein levels in exhaled breath samples during the use of four e-liquids in an unbranded EVP. We selected these constituents because nicotine, propylene glycol, glycerin, and menthol are major ingredients in the e-liquid, and formaldehyde, acetaldehyde, and acrolein are considered harmful and potentially harmful constituents. The in-clinic study was a single-center trial conducted in North Carolina. All EVP use and assessments occurred on-site. Compliance was monitored and recorded by site personnel and representatives from the contract research organization monitoring the study (Cato Research, Durham, NC). The study was conducted in accordance with Good Clinical Practice (GCP) based on the International Conference on Harmonization guidelines for GCP, and the corresponding sections of the United States Code of Federal Regulations (CFR) governing the Protection of Human Subjects (21 CFR 50), Institutional Review Boards (IRBs; 21 CFR 56), and the Basic Principles of the Declaration of Helsinki. Prior to the start of the study, the protocol and informed consent form were approved by the Chesapeake IRB (Columbia, MD; now Advarra IRB); OHRP/FDA IRB Registration Number: IRB#00,000,790; protocol approval: Pro00020903 (initial). Subsequent modifications each have a unique number (MOD00201162, MOD00202577). All study participants signed an informed consent prior before starting the study conduct.

Subjects were randomly assigned by sex to one of four use sequences in a 1:1:1:1 ratio (*N* = 8 subjects in each sequence: ABDC, BCAD, CDBA, or DACB). Subjects used one e-liquid per day in the order of their assigned sequence. The sample size was considered adequate to generate the observations regarding exhaled breath levels.

On each study day (Day 1–Day 4), subjects completed a morning exhaled breath session with their assigned e-liquid. After session completion, subjects were allowed ad libitum use of their assigned e-liquid with new cartridges and freshly charged batteries for the next 12 h with puff topography assessment (data not shown). Subjects underwent end-of-study assessments and were released from the site upon completion of the ad libitum use session on Day 4.

### Participant selection

Subject candidates were required to provide voluntary consent to participate and meet certain criteria before study enrollment: adults (21–65 years of age, inclusive), generally healthy, users of nicotine-containing EVPs for at least 3 months, users of nicotine-containing EVPs (some days or every day) for the past 30 days and at least 4 out of the past 7 days, and a positive urine test for tobacco use. All subjects had to be willing to use the EVPs after a brief test trial on Day 1 that consisted of ad libitum use for 10 min with each test e-liquid separated by ~ 30 min from the end of one trial to the start of another. Subjects who agreed to comply with the study procedures and met all other inclusion criteria and no exclusion criteria were eligible to participate. Eligible subjects checked in to the clinic the day before the exhaled breath collections started and were confined to the clinic for the remainder of the study.

### Exhaled breath sample collection and analyses

Each exhaled breath session consisted of: (1) sample collection during use of an EVP with an empty cartridge and an inactive battery (sham condition); (2) sample collection using the assigned e-liquid with all exhaled breath collected in Trapping Container One (Collection 1, which captured nicotine, propylene glycol, glycerin, and menthol) for both sham and EVP collections; (3) at least 45 min of rest; (4) repeated sham sample collection; and (5) sample collection with Trapping Container Two (Collection 2, which captured formaldehyde, acetaldehyde, and acrolein) for both the second sham and EVP collections. Both types of trapping containers were supplied by Enthalpy Analytical, Inc. (Durham, NC). Each sample consisted of all the exhaled breaths occurring during 10 puffs, each with 5-s puff duration (± 1 s), for approximately 5 min (1 puff approximately every 30 s) collected in the respective sample collection containers. These collections took place in the morning during Day 1 through Day 4 of in-clinic confinement.

### Exhaled breath trapping containers

The exhaled breath condensate samples were collected using a fresh Single-Subject Sampling Kit provided by Enthalpy Analytical (Richmond, VA). Two trapping container systems were used with different filter configurations depending on the analytes of interest. The particulate filter for Trapping Container One consisted of a single 50-mm AirLife Bacterial/Viral filter housed in a plastic filter holder. The filter was removed from the holder and used for measurement of nicotine, propylene glycol, glycerin, and menthol. The same measurements were performed for liquid in the cryogenically cooled trap after it was removed from the exhaled breath sample collection system. The filter system for Trapping Container Two consisted of two 50-mm AirLife Bacterial/Viral filters housed in a plastic filter holder. The liquid in the cryogenically cooled trap was removed from the exhaled breath sample collection system, treated on-site with 2,4-dinitrophenylhydrazine, and analyzed for formaldehyde, acetaldehyde, and acrolein. Upon completion of sample collection, the exhaled breath sample collection systems were transported to Enthalpy Analytical for high-performance liquid and gas chromatography analyses as described in detail elsewhere [17].

### Statistical analyses

A linear mixed model for analysis of covariance (ANCOVA) was used on the sham-adjusted analyte levels in the exhaled breath samples. The model included sequence, EVP, and period as fixed effects; sham sample value as a fixed covariance; and subject nested within-sequence as a random effect. All values of analytes in the exhaled samples below the minimum detectable level (MDL) were replaced with the MDL value for that sample. All values above the MDL were used as reported.

### Sample size estimation

This study was conducted to characterize exhaled breath of subjects using the four test products. Due to the limited available data on exhaled breath during e-vapor product use, a sample size of 32 subjects is believed to be appropriate for descriptive purposes.

### Computational room air level model 

The well-mixed model is described in detail elsewhere and was previously validated with experimental data; it allows an estimation of aerosol dispersion and room air levels of individual constituents in an indoor space [18]. The model was developed to estimate room air levels of four constituents: nicotine, formaldehyde, acrolein, and acetaldehyde. The modeling approach focused on the four constituents present in the exhaled breath of EVP users, and other chemical signatures due to human presence and activities [19] were excluded because these were the only constituents that were quantifiable above the detection limits in the exhaled breath.

The room air level of individual constituents in an indoor space can be calculated using computational methods. In general, two methods are available for this purpose: a distributed model [20] and a well-mixed model [18]. A distributed model uses three-dimensional airflow and transport partial differential equations along with thermodynamic and energy equations and provides spatial and temporal profiles of the concentrations in air. This model requires knowledge of the precise location of the source of the exhaled breath as well as its time-dependent rate of release as well as the ventilation inlet and outlet location and sizes. For practical purpose of this study where these information are not specified, we used a well-mixed model. Furthermore, the distributed model results are only applicable to those specific source locations and ventilation outlets positions, while the well-mixed model results are averaged values and are not limited to those specific cases at the expense of fewer details.

A well-mixed model assumes that the room air level of constituents is the same everywhere in the room at each time, but changes with time. For this assumption to be reasonable, the room air ventilation must be high enough so that the mixing time of the constituents with air remains considerably shorter than the duration of exposure. For the examples that follow, we have arbitrary assumed high ventilation rate to make the mixing time much shorter than the exposure durations of 1–4 h. Another assumption used in this well- mixed model is that there is thermodynamic equilibrium between the particulate and vapor phases of each constituents. This assumption is easily justifiable as the heat transfer and mass transfer coefficients at particle-vapor interface are very large due to small particles sizes. A simple calculation for submicron particles shows that the time to reach particle–vapor equilibrium is under a millisecond. Finally, the EVP constituents are highly volatile as compared to cigarette smoke particles resulting in a longer residence time of smoke particles in air.

**Fig. 1:**
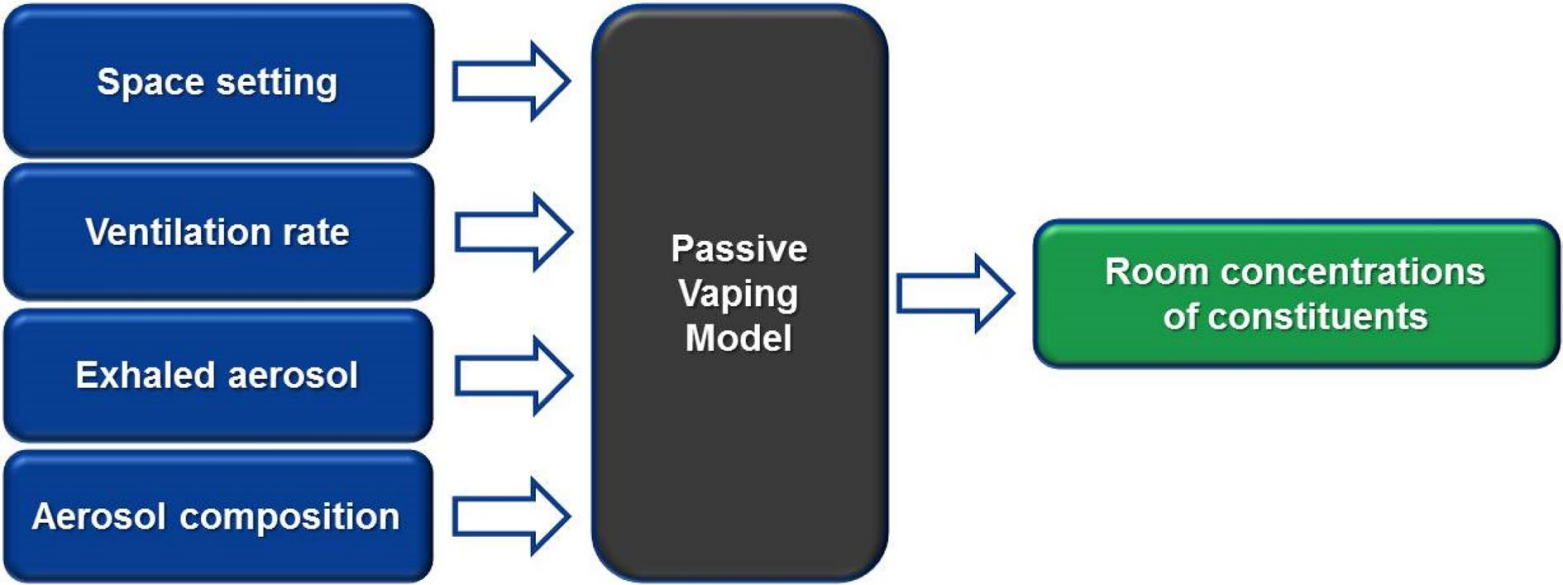
Schematic of the non-user exposure characterization models; the physics-based model considers fluid flow, mass and heat transfers, and thermodynamic and kinetic interactions

Test Product 3 was selected for modeling estimates as it yielded relatively higher formaldehyde values in exhaled breath among the test products in the study. The model predicts vapor–particle partitioning and concentrations of chemical constituents of aerosol over time, as they travel through a defined indoor space. Input variables (Fig. 1) include space setting (space type and volume such as car, office space, or a restaurant), ventilation rate (fresh air exchange rate in air change per hour), exhaled aerosol (the amount of total aerosol exhaled by all users), and aerosol composition (mass fraction of each constituent of interest in the exhaled aerosol). The model is based on physical and thermodynamic interactions between air, vapor, and the particulate phase of the aerosol. These processes are mathematically represented by a set of simultaneous equations including conservation of mass, vapor/liquid partitioning, air flow and species transport, and mixing processes [18].

Since the publication of this model [18], more data on the concentration of constituents in air have become available. In a more recent publication [21], air level of constituents from using JUUL ENDS in an environmental chamber, where the fresh air ventilation rates were varied to represent ASHRAE recommended ventilation rates corresponding to residential, office and hospitality spaces. Data for a different EVP product used in the same environmental chamber at different ventilation rates were used to validate the model [18].

The modeling approach focused on the four constituents present in the exhaled breath of EVP users and other chemical signatures due to human presence and activities [19] were excluded.

The EVP user exhaled breath results from the present study and historic sidestream smoke from a conventional combustible cigarette were entered into a well-mixed computational model to estimate concentrations of aerosol constituents in three space settings where EVPs or cigarettes are used: (1) car (3.17 m3, closed versus open [3-inch gap] windows, 2 users among 4 occupants); (2) meeting room (81 m3, 3 users among 15 occupants); and (3) restaurant (270 m3, 15 users among 100 occupants). These concentrations were compared with corresponding values from the use of conventional cigarettes as well as with the permissible exposure limits (PELs) of the Occupational Safety and Health Administration (OSHA) [22]. The prevalence of smokers among the adult population in the United States was obtained from a Centers for Disease Control report [23], and the same prevalence was assumed for EVP use. One cigarette per hour for 16 h was used for daily cigarette consumption to allow for easy 1-h exposure blocks and is similar to data on the daily consumption of cigarettes [23]. E-liquid consumption per day was based on approximately 902 mg average daily e-liquid consumption [average daily consumption for 7 days in-clinic assessment of exclusive use of Test Product 3 by smokers (16 h ad libitum use per day)] [24]. Intake by non-users was based on the assumption that 100% of inhaled analytes are absorbed and estimated as follows: average concentration × exposure duration × breathing volume × breathing rate. A breathing volume of 500 mL at a rate of 12 breaths/min was used in all calculations.

## Results

### Participant characteristics and safety

The mean age of study participants (*N*  = 35) was 35 years (range 23–62 years). A total of 20 males (57.1%) and 15 females (42.9%) were enrolled into the product trial; 68.6% of subjects were African American, 28.6% were Caucasian, and 2.9% self-reported as belonging to a race other than African American or Caucasian.

Three subjects discontinued participation before randomization. One female subject who was unwilling/unable to use all four e-liquids (on Day 1), and two additional male subjects were never randomized into the study because enrollment had been met. A total of 32 subjects were randomized and all subjects completed the study.

All four e-liquids were well-tolerated, with seven (20.0%) subjects experiencing a combined total of 12 adverse events (AEs) that were mild in severity. The most common AE was rash (three subjects experienced one event each). Only 4 of the 12 AEs were considered related to the test product by the principal investigator. These four related AEs (coughing, oral discomfort, vomiting, and dizziness) were reported by only one subject each (2.9% of total subjects, respectively). No serious AEs were reported.

### Exhaled breath

**Fig. 2:**
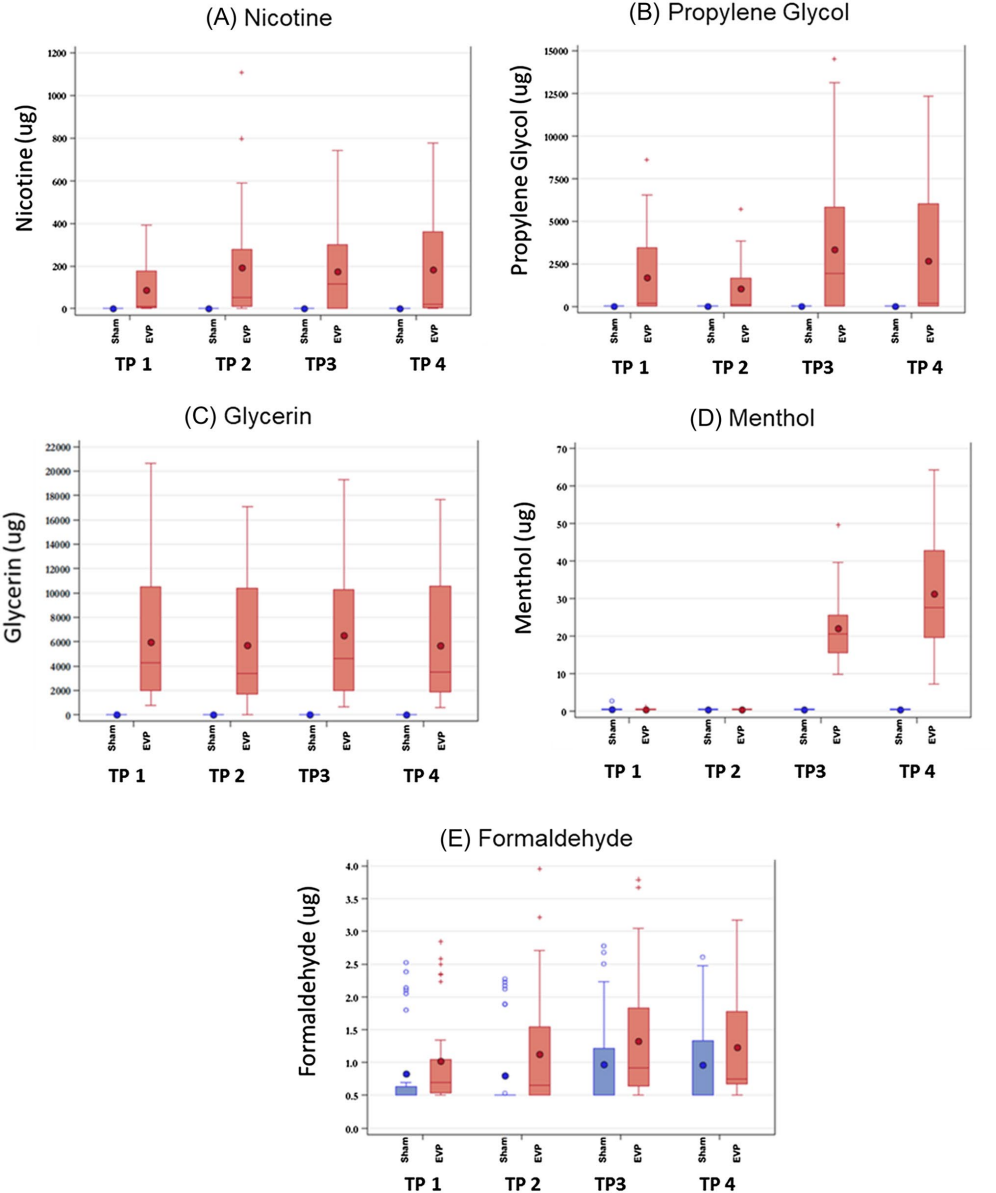
Distributions of sham and EVP exhaled breath measurements of select constituents during 10 puffs at 5 s each. Levels of nicotine (**A**), glycerin (**B**), propylene glycol (**C**), menthol (**D**), and formaldehyde (**E**). Levels below the minimum detection limit were set to the minimum detection limit. Outliers are represented by open circles (sham) and plus signs (EVP); means are represented by the closed circles; first quartiles are represented by the bottom of the boxes; medians are represented by the top of the boxes; and 1.5 times the first and third quartiles are represented by the lines extending below and above the boxes, respectively. Blue and red bars indicate sham and EVP values, respectively. *TP* test product(colour figure online)

The ranges of measured levels of each of the detected analytes were calculated using exhaled breath samples, and substantial variability was observed for each analyte (Fig. 2). Acetaldehyde and acrolein were all below the MDLs in all exhaled breath samples and therefore not analyzed further. Menthol and formaldehyde levels in exhaled breath samples of subjects were below the MDLs in 50% and 17%, respectively, of exhaled breath samples (Supplementary Table S2). We note that large variability was observed in formaldehyde measurements for both sham and test product use (Fig. 2). The average amount of e-liquid consumed during the collections ranged from 33.7 mg to 41.2 mg (Table 1). The levels of each analyte present in exhaled breath samples were analyzed using linear mixed-effects ANCOVA models. The data revealed that sham-corrected ANCOVA least square means (95% confidence interval; CI) in 10 puffs for the four most abundant analytes were significantly different from zero (Table 2). Sham-corrected ANCOVA least square means (95% CI) for menthol showed significant differences from zero only after use of menthol-flavored e-liquids (Test Products 3 and 4). The levels of any of the seven analytes in the exhaled breath of subjects were not significantly affected by study period or sequence of EVP use. Observed, sham-corrected levels of nicotine (least square means ranged from 89.44 to 195.70 µg), propylene glycol (least square means ranged from 1,199.7 to 3,354.5 µg), glycerin (least square means ranged from 5,366.8 to 6,484.7 µg), and formaldehyde (least square means ranged from 0.25 to 0.34 µg) in 10 puffs were significantly different from zero (*p* < 0.05) in subjects’ exhaled breath after use of every e-liquid. Test Products 2 and 4 contained the highest percentage NBW (4.0%) and yielded the highest nicotine levels in exhaled breath samples (195.70 and 182.65 µg, respectively). Differences in sham-corrected menthol levels were only significantly different from zero after mentholated e-liquid use (Test Products 3 and 4; 21.11 and 31.01 µg, respectively).

**Table 1:** E-liquid consumption during exhaled breath collections

Exhaled breath collection		Test product 1	Test product 2	Test product 3	Test product 4
	*N*	32	32	32	32
Collection 1 (Nicotine, propylene glycol, glycerin, menthol)	Mean (mg)	35.2	33.7	40.2	36.7
	SD	8.70	11.37	12.6	12.32
Collection 2 (Formaldehyde, acetaldehyde, acrolein)	Mean (mg)	35.6	36.3	41.2	37.9
	SD	11.53	11.94	14.58	12.4

*SD* standard deviation

**Table 2:** Summary of estimates from mixed-effects ANCOVA model for sham-corrected analytes

Analyte	E-liquid	*N*	Least square mean (95% CI) [µg]	*p*^a^
Nicotine	Test product 1	32	89.44 (15.77, 163.11)	0.0184
	Test product 2	32	195.70 (122.01, 269.38)	< 0.0001
	Test product 3	32	168.83 (95.17, 242.50)	< 0.0001
	Test product 4	32	182.65 (108.63, 256.67)	< 0.0001
Propylene glycol	Test product 1	32	1,678.4 (592.14, 2764.6)	0.0031
	Test product 2	32	1,199.7 (113.74, 2285.7)	0.0310
	Test product 3	32	3,354.5 (2266.6, 4442.3)	< 0.0001
	Test product 4	32	2,511.0 (1416.1, 3605.9)	< 0.0001
Glycerin	Test product 1	32	5,972.3 (4,191.1, 7,753.6)	< 0.0001
	Test product 2	32	6,099.5 (4,317.8, 7,881.2)	< 0.0001
	Test product 3	32	6,484.7 (4,701.7, 8,267.6)	< 0.0001
	Test product 4	32	5,366.8 (3,575.9, 7,157.7)	< 0.0001
Menthol	Test product 1	32	0.17 (–2.92, 3.25)	0.9150
	Test product 2	32	0.35 (–2.70, 3.40)	0.8217
	Test product 3	32	21.11 (18.06, 24.16)	< 0.0001
	Test product 4	32	31.01 (27.91,34.12)	< 0.0001
Formaldehyde	Test product 1	32	0.25 (0.12, 0.38)	0.0002
	Test product 2	32	0.25 (0.12, 0.38)	0.0003
	Test product 3	32	0.34 (0.21, 0.47)	< 0.0001
	Test product 4	32	0.30 (0.17, 0.43)	< 0.0001

*ANCOVA* analysis of covariance, *CI* confidence interval

^a^Test whether the sham-corrected value is different than 0

### Modeling room air levels and non-user intake

The sham-corrected exhaled breath concentrations were used as inputs to a computational room air level model (Fig. 1). The input data for the room air level modeling are outlined in Table 3, and the estimated room air levels for four space settings are available in Supplementary Table S3. The estimated intake by non-users in the space settings shared with cigarette smokers or EVP users are listed in Table 4.

**Table 3:** Data used for input into non-user intake model during use of test product 3

Constituent	Conventional cigarette sidestream emission^a^ (µg per cigarette)	Amount exhaled^b^ per mg e-liquid consumed^c^ (µg)
Nicotine	5600	4.22
Propylene glycol	NA	83.86
Glycerin	NA	162.12
Menthol	NA	0.53
Formaldehyde	700	0.0083
Acetaldehyde	4200	0^d^
Acrolein	1300	0^d^

^a^Sidestream values from a Kentucky reference 1R4F cigarette

^b^Results from analysis of covariance (ANCOVA) analysis

^c^40 mg used for collection 1, and 41 mg used for collection 2 (rounded test product 3 data; Table 1)

^d^Below the minimum detectable level in exhaled breath collections for both sham and after EVP use

**Table 4:** Estimated intake^a^ by non-users

Test space	Duration (h)	Intake after cigarette use (µg)^b^	Intake after EVP use (µg)^c^	Intake based on 8-h exposure to OSHA PELs (µg)
Nicotine				
Car (closed windows)	1	50.95	2.07	1440
Car (open windows)	1	24.37	1.01	
Meeting room	4	158.6	6.57	
Restaurant	2	41.39	1.75	
Propylene glycol				
Car (closed windows)	1	N/A	41.31	28,800
Car (open windows)	1	N/A	20.19	
Meeting room	4	N/A	130.56	
Restaurant	2	N/A	34.95	
Glycerin				
Car (closed windows)	1	N/A	79.85	28,800
Car (open windows)	1	N/A	39.04	
Meeting room	4	N/A	252.40	
Restaurant	2	N/A	67.57	
Formaldehyde				
Car (closed windows)	1	6.36	0.00408	2650
Car (open windows)	1	3.04	0.00199	
Meeting room	4	19.83	0.01291	
Restaurant	2	5.17	0.00345	
Acetaldehyde				
Car (closed windows)	1	12.28	0	1,036,800
Car (open windows)	1	5.88	0	
Meeting room	4	38.25	0	
Restaurant	2	9.98	0	
Acrolein				
Car (closed windows)	1	38.2	0	720
Car (open windows)	1	18.29	0	
Meeting room	4	119.00	0	
Restaurant	2	31.05	0	

^a^Intake = (average concentration) × (exposure duration) × (breathing volume) × (breathing rate)

^b^N/A refers to the cases where no data are available for that constituent in sidestream smoke for comparison

^c^Zero values represent cases where the measured level of the constituent was below the minimum detectable level

The results indicate that non-user intake of nicotine and formaldehyde, would occur, but at substantially lower levels during EVP use compared with secondhand exposure to conventional cigarettes (Table 4). Acetaldehyde and acrolein were not detected in exhaled breath during EVP use under these study conditions, thus no estimated exposure to non-EVP users is reported. No data for propylene glycol or glycerin in sidestream smoke were available to compare with the EVP case. However, room-level measurements of constituents show that the average concentrations of propylene glycol in a room where cigarettes and EVPs were used ad libitum were 66 and 132 µg/m3, respectively [5]. The respective values for glycerin in that study were below the level of quantification and 78 µg/m3 for cigarettes and EVPs, respectively [5].

For reference, the modeled indoor air levels were compared to the OSHA PELs, defined as the limit of the total average airborne exposure in any 8-h work shift of a 40-h work week. The OSHA PELs [22] are as follows: nicotine, 500 µg/m3; glycerin, 10,000 µg/m3; formaldehyde 920 µg/m3; acetaldehyde, 360,000 µg/m3; and acrolein, 250 µg/m3. Computational modeling showed that analyte concentrations in air after EVP use for all modeled indoor spaces (Table 4 and Supplementary Table S3) were orders of magnitude less than the OSHA PELs for an 8-h workday.

### Discussion

The key findings from the exhaled breath assessment were: (1) samples from all subjects were below the MDLs for acetaldehyde and acrolein after EVP use; (2) menthol was detected only in the two mentholated e-liquids; (3) nicotine, glycerin, propylene glycol, and formaldehyde were detected in the exhaled breath of subjects for all four e-liquids; and, (4) significant variability existed between subjects in the levels of analytes in exhaled breath, despite subjects using prespecified puffing conditions. These data from real users were entered into a well-mixed model to estimate potential secondhand exposure. The key findings from the computational model were: (1) the computational model is fit-for-purpose to predict constituent levels under various real-world scenarios; (2) room air levels of nicotine, formaldehyde, acrolein, and acetaldehyde levels were significantly below OSHA PELs or American Industrial Hygiene Association (AIHA) limit; and (3) intake of these constituents by non-users would be substantially lower in the presence of EVP use compared with secondhand exposure to conventional combustible cigarettes.

These findings are consistent with previous machine puffing and exhaled breath studies that showed wide variabilities in analyte levels across different EVPs [13, 14]. Exhaled breath or aerosols from such studies consistently showed that nicotine, propylene glycol, and glycerin were present in higher concentrations than the other analytes characterized in the current study since propylene glycol and glycerin are the main nicotine carriers in e-liquids. Similar to our findings, another group reported that formaldehyde levels were low and that acetaldehyde and acrolein were undetectable in various brands of EVPs [14]. However, others measured detectable levels of these compounds depending on the device and e-liquid used [9, 25]. In a more recent publication [24], air level of constituents from using another EVP that is currently available in the marketplace (JUUL) in an environmental chamber, where the fresh air ventilation rates were varied to represent ASHRAE recommended ventilation rates corresponding to residential, office and hospitality spaces. Our findings complement the observations reported by Oldham et al. [24]. In addition, the well-mixed model results are similar to actual room air measurements reported by others, under scenarios of EVP use in passenger cars and a small room [26, 27]. Schober et al. (2019) report that actual values during a 20-min use occasion in a passenger car ranged from 50 to 762 µg/m3 for propylene glycol, and nicotine levels ranged from below detection limit up to 10 µg/m3 for nicotine [27]. These values observed under actual use of EVPs are well within the ranges predicted from our model (56.09–114.74 µg/m3 for propylene glycol and 2.82–5.77 µg/m3 for nicotine, Table S3a and b). Overall, these comparisons to published literature demonstrate the applicability of this validated model to different “real-world” scenarios.

The main finding from the well-mixed modeling was the establishment of a fit-for-purpose computational model to predict room air levels under various real-world scenarios. The test space concentrations of nicotine, formaldehyde, acetaldehyde, and acrolein were significantly less with EVPs compared to cigarettes under equivalent use conditions. Propylene glycol and glycerin levels in air from EVP use were orders of magnitude less than OSHA PELs and the AIHA limit for all studied spaces. The well-mixed model findings were used to estimate exposure to non-users. The predicted nicotine exposure was roughly 20-fold lower for EVP use versus cigarettes for all space settings. The difference in estimated formaldehyde exposure was even more dramatic; predicted intake by non-users ranged from 3.04 to 19.83 µg for cigarettes compared to 0.002–0.013 µg for EVPs (~ 1500-fold difference). These results should also be considered in the context of levels of formaldehyde measured when humans are present in an enclosed environment which increases with some recreational and daily living activities [19]. The formaldehyde range is comparable to that reported by Visser and colleagues, who modeled non-user exposure in two scenarios [12]. We note that Visser et al. relied on a conservative estimate of 30–40% retention in the lungs, a value which contradicts the observations of > 90% retention (at least for nicotine) reported by St. Helens et al. [28]. Pulmonary retention of formaldehyde while not reported for humans, dog studies indicate almost 100% being retained in the lungs of dogs [29], suggesting that low levels of formaldehyde would likely to be exhaled after EVP use. Our nicotine range was higher, possibly because the EVPs in their study had lower NBW values and/or their modeling was based on 5 puffs instead of 10. The relevance of our findings should be considered in the context of actual exposure that might be experienced by non-users in an environment where EVPs are used. For example, Picavet et al. [30] report that nicotine exposure, as measured by urinary excretion levels, among non-smokers passively exposed to a heated tobacco product aerosol was not increased relative to those non-smokers that were not exposed. Overall, our results are in concordance with previous reports that demonstrate while EVPs use may expose non-users to some secondhand constituents, they do not substantially increase non-user exposure to combustion toxicants [12, 31, 32].

We note that there is an ongoing debate regarding the appropriateness of using the PEL of OSHA for some constituents [33]. As described by Hubbs et al. [33], the occupational exposure values represent the upper limit values that are not expected to adversely affect workers’ health over their working lives (8 h/day, 5 days/week) and do not specifically include any susceptible sub-groups or populations. Nonetheless, the comparisons to these limits set by authoritative bodies of experts are indeed applicable because they indicate the “safety limit” of exposure to the constituents in humans. The PEL of OSHA is a conservative limit assuming exposure in every breath over the 8-h period in the occupational setting. In contrast, non-users are not likely to experience such constant exposure in every breath. EVP users only intermittently use the products; thus, non-users would be likely to experience only transient exposure to the exhaled aerosol. This transient exposure is not likely to exceed the cumulative constant exposure experienced through occupational exposure. Moreover, due to the lack of other well-accepted comparative measures of exposure, and considering the basic toxicology principle of dose response, we believe that comparisons to OSHA limits provide useful insight on the level of exposure estimated under these use conditions.

Data from this study allowed for comparison of various analyte levels in the four e-liquids and estimation of ambient analyte concentrations under various EVP use scenarios. User behavior, EVP characteristics, and the dimensions and ventilation of a space all influence air concentrations. The highest predicted non-user intake was for our meeting room scenario where three people were using EVPs during a 4-h period. This is an extreme example, but expected intakes were still dramatically lower than OSHA PELs. The lowest values were in a car with open windows, which is not surprising, likely due to cross ventilation through the open windows. While conventional cigarette smokers open the windows out of necessity, EVP users may not do so. Our modeling suggests that non-user exposure could be halved if the car windows are 3 inches open during EVP use. Importantly, predicted non-user intake of formaldehyde and nicotine were several-fold lower than for conventional cigarettes.

Only a few studies have investigated secondhand exposure to EVP analytes using exhaled breath samples, and to our knowledge, the current study included the largest number of users. There were, however, several limitations to this study: (1) The subjects were instructed to take 5-s puffs with a fresh cartridge, and this might not be their usual puff duration or reflect the entire use of a cartridge; (2) the study only tested a limited number of analytes in four different flavors of the same cartridge-based EVP and, therefore, did not comprehensively characterize the values of the various analytes in the wide, fast-growing array of EVPs; and (3) the degree of passive exposure depends on multiple factors such as specific product and how it is used, ventilation rate, space size, humidity, and number of users, some of which were included in our model. Despite these limitations, our results show the utility of this modeling approach for studying non-user exposure.

## Conclusions

Use of any of the four e-liquids in this study resulted in significant increases versus sham in the levels of four of seven analytes in exhaled breath: nicotine, glycerin, propylene glycol, and formaldehyde. In addition, use of both mentholated e-liquids (Test Products 3 and 4) resulted in significant increases from sham in the level of menthol in exhaled breath. Acetaldehyde and acrolein were not detectable after use of any of the test products. When these data were used as inputs to a computational room air level and non-user intake model, the ambient concentrations of exhaled nicotine and formaldehyde predicted that non-user intakes were substantially reduced for test product use compared to conventional cigarette use. Collectively, the results predict that room air levels and exposure of the selected analytes to non-users were relatively low and several-fold below regulatory PELs and AIHA limit under the modeled space and use conditions. In this manuscript we describe the application of a validated computational model to predict room air levels based on exhaled breath measurements. The main finding from the well-mixed modeling was the establishment of a fit-for-purpose computational model to predict room air levels and non-user exposure under various real-world scenarios for any EVP. The principles described in this manuscript can be applied to any product and therefore the results add to the scientific knowledge and understanding of potential passive vaping under various “real-world” conditions. The computational model may be useful in assessing room air levels of constituents among different types of EVPs and estimating potential secondhand EVP exposure under various real-world settings.

## Supplementary Information

Below is the link to the electronic supplementary material.

Supplementary file1 (DOCX 29 KB)

## Data Availability

The datasets generated during and/or analyzed during the current study and included in this manuscript will be made available from the corresponding author on reasonable request.

## Abbreviations

AE: Adverse event
AIHA: American Industrial Hygiene Association
ANCOVA: Analysis of covariance
CFR: Code of Federal Regulation
GCP: Good Clinical Practice
IRB: Institutional Review Board
MDL: Minimum detectable level
NBW: Nicotine by weight (%)
OSHA: Occupation Safety and Health Administration
PEL: Permissible exposure limit
TP: Test product
