# Supplementary material for: Computational modeling method to estimate secondhand exposure potential from exhalations during e-vapor product use under various real-world scenarios
Source: Intern Emerg Med. 2022 Sep 1;17(7):2005–16. doi: 10.1007/s11739-022-03061-2 (PMC9522680; doi:10.1007/s11739-022-03061-2)
Supplement: Supplementary file 1 — Supplementary file1 (DOCX 29 KB) [file 11739_2022_3061_MOESM1_ESM.docx]

Supplemental Table S1. EVP emissions of analytes during machine puffing^A^.

| **EVP** | **Statistic** | **Nicotine (µg /puff)** | **Propylene glycol (µg/puff)** | **Glycerin (µg/puff)** | **Menthol (µg/puff)** | **Formaldehyde (µg/puff)** | **Acetaldehyde (µg/puff)** | **Acrolein (µg/puff)** |
| --- | --- | --- | --- | --- | --- | --- | --- | --- |
| Test Product 1 | Mean | 126 | 1,470 | 2,521 | BLOQ | 0.100 | 0.065 | BLOQ |
|  | SD | 6 | 74 | 123 | – | 0.029 | 0.008 | – |
| Test Product 2 | Mean | 201 | 1,011 | 2,885 | BLOQ | 0.118 | 0.090 | BLOQ |
|  | SD | 13 | 42 | 97 | – | 0.017 | 0.008 | – |
| Test Product 3 | Mean | 187 | 1,986 | 2,240 | 81 | 0.187 | 0.075 | BLOQ |
|  | SD | 5 | 70 | 75 | 3 | 0.113 | 0.048 | – |
| Test Product 4 | Mean | 232 | 1,940 | 2,294 | 139 | 0.133 | 0.051 | BLOQ |
|  | SD | 24 | 245 | 311 | 14 | 0.023 | 0.006 | – |

BLOQ = below limit of quantification (0.05 mg menthol per puff and 0.016 µg acrolein per puff)

^A^ Cartridge weight changes for the four EVPs ranged from 5.267-6.088 (SDs 0.177-0.603).

Supplementary Table S2. Summary of distribution below MDL of sham versus EVP cartridges by analyte.

| **Analyte** | **MDLs (µg)** | **Cartridge type** | **Total N** | **Samples below MDL** |
| --- | --- | --- | --- | --- |
| Nicotine | 0.3380A or 1.6280B | Sham | 128 | 118 (92.2%) |
|  |  | EVP | 127 | 12 (9.4%) |
| Propylene glycol | 14.5000 | Sham | 128 | 121 (94.5%) |
|  |  | EVP | 127 | 5 (3.9%) |
| Glycerin | 10.0600 | Sham | 128 | 112 (87.5%) |
|  |  | EVP | 127 | 1 (0.8%) |
| Menthol | 0.3300a or 0.4820b | Sham | 128 | 127 (99.2%) |
|  |  | EVP | 127 | 63 (49.6%) |
| Formaldehyde | 0.5000 | Sham | 128 | 83 (64.8%) |
|  |  | EVP | 128 | 22 (17.2%) |
| Acetaldehyde | 0.4975 | Sham | 128 | 128 (100.0%) |
|  |  | EVP | 128 | 128 (100.0%) |
| Acrolein | 0.6270 | Sham | 128 | 128 (100.0%) |
|  |  | EVP | 128 | 128 (100.0%) |

MDL = minimum detectable level

^A^ Assays performed on all dates except 27 March 2017 and 01 April 2017

^B^ Assays performed on 27 March 2017 and 01 April 2017

Supplementary Table S3a. Average nicotine concentration in air (µg/m^3^).

|  | **Cigarette** | **Test Product 3** | **OSHA PEL** |
| --- | --- | --- | --- |
| Car (closed windows) | 141.54 | 5.77 | 500 |
| Car (open windows) | 67.71 | 2.82 | 500 |
| Meeting room | 126.53 | 5.36 | 500 |
| Restaurant | 64.00 | 2.71 | 500 |

OSHA = Occupational Safety and Health Administration; PEL = permissible exposure limit.

Supplementary Table S3b. Average propylene glycol concentration in air (µg/m^3^).

|  | **Cigarette** | **Test Product 3** | **AIHA Limit** |
| --- | --- | --- | --- |
| Car (closed windows) | N/A | 114.74 | 10,000 |
| Car (open windows) | N/A | 56.09 | 10,000 |
| Meeting room | N/A | 106.59 | 10,000 |
| Restaurant | N/A | 54.03 | 10,000 |

AIHA = American Industrial Hygiene Association.

Supplementary Table S3c. Average glycerol concentration in air (µg/m^3^).

|  | **Cigarette** | **Test Product 3** | **OSHA PEL** |
| --- | --- | --- | --- |
| Car (closed windows) | N/A | 221.81 | 10,000 |
| Car (open windows) | N/A | 108.44 | 10,000 |
| Meeting room | N/A | 206.07 | 10,000 |
| Restaurant | N/A | 104.45 | 10,000 |

OSHA = Occupational Safety and Health Administration; PEL = permissible exposure limit.

Supplementary Table S3d. Average menthol concentration in air (µg/m^3^).

|  | **Cigarette** | **Test Product 3** | **OSHA PEL** |
| --- | --- | --- | --- |
| Car (closed windows) | N/A | 0.725 | N/A |
| Car (open windows) | N/A | 0.354 | N/A |
| Meeting room | N/A | 0.673 | N/A |
| Restaurant | N/A | 0.341 | N/A |

OSHA = Occupational Safety and Health Administration; PEL = permissible exposure limit.

Supplementary Table S3e. Average formaldehyde concentration in air (µg/m^3^).

|  | **Cigarette** | **Test Product 3** | **OSHA PEL** |
| --- | --- | --- | --- |
| Car (closed windows) | 17.69 | 0.0113 | 920 |
| Car (open windows) | 8.46 | 0.0055 | 920 |
| Meeting room | 15.81 | 0.0105 | 920 |
| Restaurant | 8.00 | 0.0053 | 920 |

OSHA = Occupational Safety and Health Administration; PEL = permissible exposure limit.

Supplementary Table S3f. Average acetaldehyde concentration in air (µg/m^3^).

|  | **Cigarette** | **Test Product 3** | **OSHA PEL** |
| --- | --- | --- | --- |
| Car (closed windows) | 34.12 | NA – input was below detection limits | 360,000 |
| Car (open windows) | 16.32 | NA – input was below detection limits | 360,000 |
| Meeting room | 30.50 | NA – input was below detection limits | 360,000 |
| Restaurant | 15.42 | NA – input was below detection limits | 360,000 |

OSHA = Occupational Safety and Health Administration; PEL = permissible exposure limit.

Supplementary Table S3g. Average acrolein concentration in air (µg/m^3^).

|  | **Cigarette** | **Test Product 3** | **OSHA PEL** |
| --- | --- | --- | --- |
| Car (closed windows) | 106.15 | NA – input was below detection limits | 250 |
| Car (open windows) | 50.804 | NA – input was below detection limits | 250 |
| Meeting room | 94.90 | NA – input was below detection limits | 250 |
| Restaurant | 48.00 | NA – input was below detection limits | 250 |

OSHA = Occupational Safety and Health Administration; PEL = permissible exposure limit.
